# Molecular Diagnosis to Individualized Therapies in Rare Genetic Diseases: New Approach Methodologies, RNA Therapeutics, and the Case for a Human-First Filter

**DOI:** 10.3390/genes17070780

**Published:** 2026-07-03

**Authors:** Saeed Anwar, Toshifumi Yokota

**Affiliations:** 1 Department of Medical Genetics, Faculty of Medicine and Dentistry, University of Alberta, Edmonton, AB T6G 2H7, Canada; 2 Neuroscience and Mental Health Institute, Faculty of Medicine and Dentistry, University of Alberta, Edmonton, AB T6G 2E1, Canada; 3 Cardiovascular Research Institute, Faculty of Medicine and Dentistry, University of Alberta, Edmonton, AB T6G 1C9, Canada; 4 Women and Children’s Health Research Institute, Faculty of Medicine and Dentistry, University of Alberta, Edmonton, AB T6G 1C9, Canada; 5 The Friends of Garrett Cumming Research and Muscular Dystrophy Canada Endowed Research, University of Alberta, Edmonton, AB T6G 2H7, Canada

**Keywords:** new approach methodologies (NAMs), RNA therapeutics, antisense oligonucleotides (ASOs), small interfering RNAs (siRNAs), rare genetic diseases, pharmacokinetics/pharmacodynamics (PK/PD), clinical-trials-in-a-dish (CTiD), induced pluripotent stem cells (iPSCs), engineered tissues, microphysiological systems

## Abstract

Rare genetic diseases are heterogeneous across mechanisms, trajectories, and treatment responses. To date, approved therapies remain available for only a small proportion of rare genetic diseases. Oligonucleotide-based RNA therapeutics, particularly antisense oligonucleotides (ASOs) and small interfering RNAs (siRNAs), offer a promising therapeutic avenue for rare genetic diseases with sequence-level precision. However, traditional preclinical paths may mis-predict human outcomes when disease biology diverges from animal models. New approach methodologies (NAMs), including patient-derived induced pluripotent stem cells (iPSCs), organoid models, and clinical-trials-in-a-dish (CTiD), aim to bring human biology earlier into the translational pipeline. NAMs enable variant-to-function studies, efficacy screening, and safety triage at clinically relevant speed and scale. While critics argue that NAMs are unvalidated and cannot replace preclinical animal models, proponents report that they are increasingly able to recapitulate human phenotypes and predict clinical liabilities, although their predictive validity remains context-dependent. Here, a front-loaded human filter refers to the use of human-derived systems early in development to support mechanistic interpretation, candidate prioritization, and early liability assessment before broader nonclinical evaluation. Recent studies pairing NAMs with ASOs support rapid, patient-specific preclinical screening in selected settings, while also showing the need for broader evidence on delivery, pharmacology, safety, and clinical relevance. This review places these developments within the translational realities of oligonucleotide-based therapeutics, including model fidelity, ASO chemistry and optimization, delivery challenges, pharmacology, regulatory pathways for individualized ASOs, and accessibility. We also propose a pragmatic validation framework to assess the scientific and translational credibility of NAMs across rare genetic diseases.

## 1. Introduction

Rare diseases are characterized by low prevalence, usually in the range of 1 in 2000 (EU) to 1 in 200,000 (US) people [1,2]. These conditions are often chronic, progressive, and, in more than 70% of cases, genetic [3,4]. While individually rare, they collectively constitute a major unmet medical need. Current estimates frequently cite several thousand distinct rare diseases, typically between 7000 and 10,000, and place the global affected population at more than 300 million [1,5]. However, treatment availability remains limited. Despite regulatory incentives, e.g., the US Orphan Drug Act, only a small proportion of rare diseases have an approved therapy, commonly estimated in the range of 5% to 10% [1]. In fact, for many patients, the path to an accurate diagnosis, if there are any, remains prolonged and difficult to access. Reports suggest that average diagnostic intervals often extend over several years, with substantial heterogeneity across regions, age groups, and disease categories [6].

Advances in genomic sequencing technology over the past couple of decades have significantly improved the diagnostic landscape for rare genetic diseases. Clinical exome sequencing established a practical and scalable route to molecular diagnosis in suspected Mendelian genetic disorders, with diagnostic yields typically reported in the range of 20–40%, and systematic reanalysis and trio or genome sequencing further increase yield and re-solve many previously unsolved cases [7,8,9,10,11]. Even then, molecular diagnosis does not always reliably translate into therapeutic action. Identification of a pathogenic variant underlying a disease can clarify disease mechanism and can help define precision therapeutic opportunities for that particular condition; however, the interval from variant discovery to an actionable, regulatory-approved intervention remains long and highly uncertain [1].

This gap makes rare genetic diseases a particularly important setting for precision medicine. Many rare disorders are monogenic or genetically stratifiable and stem from definable transcript abnormalities, e.g., splicing defects or toxic alleles. RNA therapeutics are especially attractive in this context. Antisense oligonucleotides (ASOs) and small interfering RNAs (siRNAs) offer sequence-directed, non-permanent ways to modulate transcripts [12,13,14,15]. Among these modalities, ASOs in particular can be carefully designed to alter splicing or reduce pathogenic transcripts in a mechanism-directed fashion [14]. Growing regulatory attention to individualized ASOs and other individualized therapies further indicates that rare-disease precision medicine is now being approached as a practical translational problem rather than only as a conceptual goal [16,17,18,19]. This translational challenge is inseparable from the choice of model. Traditional preclinical pipelines rely heavily on animal models. These models remain essential for systemic pharmacology and toxicology. However, they often fail to reproduce human biology, physiology, or treatment response with sufficient fidelity [20,21,22,23,24,25,26,27,28,29]. These inherent species-specific differences become especially important when therapeutic strategies target human genomic sequence features or human-specific transcript processing events. In this context, new approach methodologies (NAMs) have gained traction as approaches that bring human biology earlier into the translational pipeline [30,31]. Although explicitly referred to as “new,” NAMs do not necessarily refer only to newly developed methods. In many cases, what is new is their regulatory use or their application in place of traditional testing requirements [32,33,34,35]. In current regulatory usage, NAMs is referred to as an umbrella term that includes human cell-based systems, organoids, micro-physiological platforms, computational models, and related approaches intended to improve the predictive value of preclinical evidence.

These platforms are not self-validating, and they are not uniformly mature. Concerns about incomplete validation, developmental immaturity, variable reproducibility, and limited representation of organism-level pharmacology remain substantial [36,37,38,39]. Simultaneously, their potential value in mechanistic clarification, candidate prioritization, and early translational judgment is increasingly difficult to ignore [40]. The most practical question, therefore, is not whether NAMs should replace animal models in the abstract, but whether they can serve as a front-loaded human filter for specific decisions, particularly in the context of developing RNA-based therapeutic modalities targeting rare genetic diseases. This write-up aims to shed light upon that question with particular emphasis on RNA-based therapeutic modalities. We sought to consider both what NAMs can already contribute and what would be required for their use to become scientifically and translationally credible.

## 2. From Molecular Diagnosis to Therapeutic Opportunity in Rare Genetic Diseases

**Figure 1:**
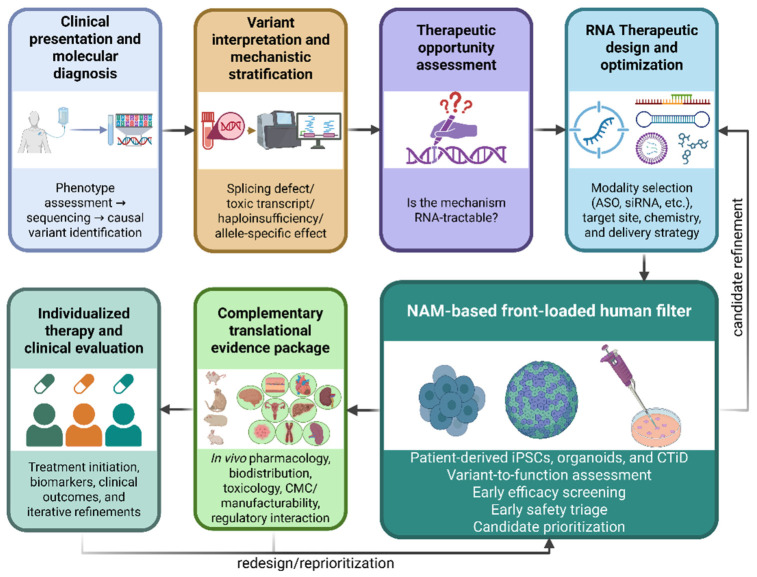
From molecular diagnosis to individualized therapy in rare genetic disease: NAMs as a front-loaded human filter. Precision medicine for rare diseases begins with molecular diagnosis and mechanistic stratification of the underlying causal variant. If the pathogenic mechanism is RNA-tractable, this information can support design of a candidate RNA-based therapeutic. NAMs, including patient-derived induced pluripotent stem cells (iPSCs), organoids, and clinical-trials-in-a-dish (CTiD) platforms, can then function as a front-loaded human filter to support mechanistic confirmation, candidate prioritization, and early liability assessment. These data may strengthen translational judgment; however, they do not replace broader evidence generation. Candidate therapies must still proceed through complementary translational steps, including in vivo pharmacology, biodistribution, toxicology, chemistry-manufacturing-controls considerations, and regulatory interaction, before individualized clinical implementation. The process is iterative, with findings from human-relevant and downstream translational studies informing re-design and re-prioritization. Colors distinguish the main workflow modules and do not encode quantitative values.

Advances in genomic technologies have substantially improved rare-disease diagnosis [41,42]. The ability to identify causal variants has increased over time, but the ability to act on those findings therapeutically has advanced more slowly [17,41]. This creates a central paradox in rare genetic disease medicine, in which diagnosis has become increasingly precise, whereas treatment remains unavailable for most patients [17,43]. However, for many rare genetic diseases, molecular diagnosis does more than only assigning a label. It can define the pathogenic mechanism with sufficient precision to suggest a therapeutic strategy [44,45]. This is especially true in monogenic disorders involving aberrant splicing, toxic gain-of-function transcripts, haploinsufficiency, pseudoexon inclusion, or allele-specific pathogenic variation [17,43,44,46,47,48]. In such settings, RNA therapeutics are particularly attractive because they operate at the transcript level and can, in principle, be matched to the disease mechanism [46,47]. ASOs can be designed to modulate splicing, suppress toxic transcripts, restore reading frame, or selectively reduce mutant RNA [46,47,48,49]. More broadly, RNA-targeted approaches offer a degree of sequence-guided adaptability that is unusual among therapeutic platforms [12,17,47]. This conceptual path, from molecular diagnosis to therapeutic nomination, is illustrated in Figure 1. Where the pathogenic mechanism is RNA-tractable, molecular diagnosis can support rational therapeutic design [44,45]. Human-derived systems can then be used upstream in the translational pathway to test whether that therapeutic hypothesis remains biologically plausible in a disease-relevant context, before broader nonclinical evaluation is pursued [31,45,50].

This logic has already been translated into early clinical and preclinical examples. The development of milasen for a child with *CLN7* Batten disease remains a landmark example in which molecular diagnosis directly informed the design and manufacture of a patient-customized splice-modulating ASO [17,44]. More recent organoid-based studies have shown that patient-derived systems can support preclinical evaluation of personalized ASOs and can reverse disease-associated phenotypes in selected settings. These examples suggest that, under some conditions, the interval between variant discovery and therapeutic nomination can be shortened substantially [44,45]. That said, molecular diagnosis does not automatically create and/or nominate therapeutic opportunity. Not every variant is mechanistically tractable at the RNA level, and not every tractable mechanism is clinically actionable. Suitability depends on several intersecting variables, including variant class, disease mechanism, target tissue, route of delivery, developmental timing, reversibility of pathology, and the availability of meaningful outcome measures [16,17]. Even within apparently ASO-responsive disease categories, translational potential can be overstated if these constraints are not considered early [17]. Rare-disease precision medicine therefore requires more than genomic resolution alone. It also requires a framework for determining whether a molecular finding is therapeutically actionable in a specific biological and clinical context [16,17,41]. That need is increasingly reflected in the regulatory landscape. Individualized ASO development is now being discussed within formal regulatory guidance for severely debilitating or life-threatening genetic diseases in which treatment-amenable variants may occur in only one or a few patients [16,17,18]. These developments acknowledge an important translational reality in ultra-rare disease, for which evidence generation may need to begin from mechanism, patient specificity, and fit-for-purpose modelling rather than from conventional population-scale development logic [16,44,45].

As such, the challenge is not simply to diagnose rare genetic disease more accurately, but to connect diagnosis to intervention more credibly [17]. That connection requires systems capable of linking genotype to cellular mechanism, mechanism to therapeutic response, and response to evidence suitable for clinical interpretation within useful timeframes [45]. RNA therapeutics make this problem especially visible because they are both modular and highly mechanism-dependent [47]. For that reason, they provide a particularly informative setting in which to examine whether human-centered preclinical systems can function as a meaningful filter between molecular diagnosis and individualized therapy [45].

## 3. RNA Therapeutics as a Precision Platform for Rare Genetic Diseases

RNA therapeutics are especially well suited to rare genetic diseases because many of these disorders are monogenic and mechanistically traceable to an abnormal transcript, a splicing defect, or a pathogenic allele that can be targeted in a sequence-directed manner [51,52,53]. This creates a strategic advantage, as unlike the conventional small molecule-based strategies, which often rely on the availability of a tractable protein target, oligonucleotide-based therapies can be designed directly against RNA and adapted to the underlying molecular lesion [52,53]. As such, RNA therapeutics provide a platform through which molecular diagnosis may, at least in selected settings, be translated into mechanism-based intervention [51,53,54]. In this review, the term RNA therapeutics is used primarily to refer to oligonucleotide-based modalities, especially ASOs and siRNAs, rather than mRNA replacement or mRNA vaccine platforms, unless explicitly stated.

Among RNA-based modalities, ASOs remain the clearest rare-disease exemplar [51]. Their versatility is central to that role. ASOs can induce RNase H-mediated transcript degradation, block or re-direct splicing, restore reading frame, suppress toxic gain-of-function transcripts, or, in selected contexts, achieve allele-selective silencing. Small interfering RNAs provide a related but distinct route to transcript knockdown through the endogenous RNA interference machinery and have shown particular translational strength in tissues with favorable delivery characteristics, especially the liver [55]. The clinical landscape now includes approved splice-modulating and transcript-silencing therapies, indicating that RNA therapeutics can be matched to disease biology with a degree of specificity that is unusual in rare disease pharmacology [53]. Some but not all examples include nusinersen for spinal muscular atrophy (SMA), exon skipping therapies for Duchenne muscular dystrophy (DMD), patisiran for hereditary transthyretin-mediated amyloidosis (hATTR), and lumasiran for primary hyperoxaluria type 1 [56,57,58,59,60,61,62,63,64,65,66,67,68].

At the same time, mechanistic elegance does not guarantee translational success [52]. Suitability depends on disease biology, target tissue, treatment window, and the chemistry and delivery strategy required to reach the relevant cells [54]. Some of the most successful RNA therapies have benefited from relatively favorable delivery contexts, e.g., intrathecal administration to the central nervous system or GalNAc-enabled targeting to hepatocytes [12,51,55]. By contrast, broad and efficient delivery to tissues, e.g., heart and skeletal muscle, remain substantially more difficult [12,69,70,71,72]. Off-target effects, sequence-dependent liabilities, class-related toxicities, and uncertainty around long-term pharmacology further complicate development [52]. As a result, the central question in rare disease is often not whether a transcript can be targeted in principle, but whether a specific RNA therapeutic can reach the right tissue, engage the intended mechanism, and produce interpretable benefit within a clinically meaningful timeframe [54].

These features make RNA therapeutics unusually informative for evaluating the broader promise of precision medicine in rare genetic diseases [52]. Their design is modular, but their success is highly context-dependent [53]. Their biological rationale is often strong, but their preclinical development can fail if the model system does not capture the relevant transcript biology, cellular phenotype, or treatment response [45]. This tension is one reason RNA therapeutics have become a major proving ground for patient-specific development. The individualized ASO field has shown that mechanism-based therapeutic design can proceed on highly compressed timelines [17]. More recent patient-derived workflows suggest that preclinical screening and response assessment may be accelerated further when human-derived systems are incorporated early [45]. Taken together, RNA therapeutics provide one of the strongest available test cases for asking whether a human-first preclinical filter can improve the path from molecular diagnosis to individualized therapy [45,54].

## 4. NAMs and the Case for a Human-First Filter

As mentioned earlier, NAMs are a broad umbrella term for experimental and computational approaches intended to generate mechanistic, safety, or efficacy-relevant data with greater human relevance than conventional animal-dependent workflows [73,74,75,76]. In current regulatory usage, NAMs may include in vitro systems, in silico models, biomarkers, and selected modified in vivo approaches. For the purposes of this review, the focus is narrower and centred on patient-derived iPSCs, organoid and engineered tissue models, and related CTiD strategies, in which therapies are evaluated in human-derived systems before or alongside conventional preclinical development [77].

The recent spike in interest in NAMs has both scientific and practical reasons [76]. From a scientific perspective, many rare genetic diseases depend on human-specific transcript processing, developmental context, or cell-type vulnerability that may not be captured adequately in animal models [77]. From a practical perspective, rare-disease therapeutic development often proceeds under severe constraints of time, patient number, and available natural history data [76]. These pressures are especially relevant to RNA therapeutics, where the therapeutic logic is frequently mechanism-specific and, in some cases, patient-specific.

NAMs can move human biology earlier into the translational pathway [78]. In principle, they can link genotypes to cellular mechanisms, support variant-to-function studies, enable therapeutic screening in disease-relevant cells, and provide an early indication of whether a candidate intervention remains biologically plausible [77]. Recent patient-derived organoid studies illustrate this potential by showing that personalized ASOs can be evaluated in human disease models within clinically meaningful timeframes and, in selected settings, can reverse disease-associated phenotypes [45].

At the same time, NAMs are not automatically predictive simply because they are human-derived [76]. Model immaturity, incomplete tissue architecture, batch variability, limited pharmacokinetic context, and inadequate capture of systemic physiology remain major constraints [77]. For this reason, the most defensible role for NAMs at present is neither wholesale replacement nor rhetorical alternative [78]. Rather, it is that of a front-loaded human filter, which is a way to improve early translational judgment, improve candidate selection, and de-risk subsequent animal and early clinical studies when used within a broader evidentiary framework [77].

This framing is especially relevant in rare genetic disease. In small-N or ultra-rare settings, where conventional evidentiary pathways may be impractical, the pressure to make earlier and better-grounded translational decisions is unusually high [77]. NAMs do not resolve that pressure on their own [75,78]. They may, however, provide a more mechanism-informed and human-relevant basis for some of those decisions, provided that their limitations are made explicit and their use remains fit-for-purpose [75,78]. Recent reviews have also increasingly framed NAMs not as isolated assays, but as interoperable evidence streams in which cellular, tissue-level, and computational systems are aligned to generate cross-scale evidence for mechanistic and translational decision-making [75,78,79]. In that sense, the value of NAMs may depend less on any single platform than on how effectively complementary human-derived systems are integrated within a fit-for-purpose development pathway [77].

## 5. What NAMs Can and Cannot Do for RNA Therapeutic Development

For RNA therapeutics, the most immediate value of NAMs lies in mechanistic resolution [76,77]. Patient-derived iPSCs, organoids, and related human-derived cell-based systems can help determine whether a candidate transcript abnormality is truly disease-relevant, whether it is therapeutically tractable, and whether modulation of that transcript produces the expected biological effect in a disease-relevant context [80,81,82,83]. This is particularly useful in rare genetic diseases driven by splice defects, toxic transcripts, allele-specific effects, or cell-type-restricted vulnerabilities [44,45]. In such settings, NAMs can function as a practical bridge between molecular diagnosis and therapeutic nomination by enabling variant-to-function studies before substantial time and resources are committed to downstream development [45,54].

Model selection is critical because different systems preserve different dimensions of human biology [76,77]. Patient-specific iPSC systems are especially well suited to genetically defined disorders because they retain donor-specific variation and permit mechanistic analysis in a controlled genomic background [81,84,85]. In contrast, mature primary human cells may better preserve age-, exposure-, or context-dependent phenotypes that are (at least) partly reset during reprogramming [86,87]. For rare-disease RNA therapeutics, this means that the most informative NAM is not necessarily the most complex one, but the one whose biological state is best matched to the mechanism being interrogated.

**Figure 2:**
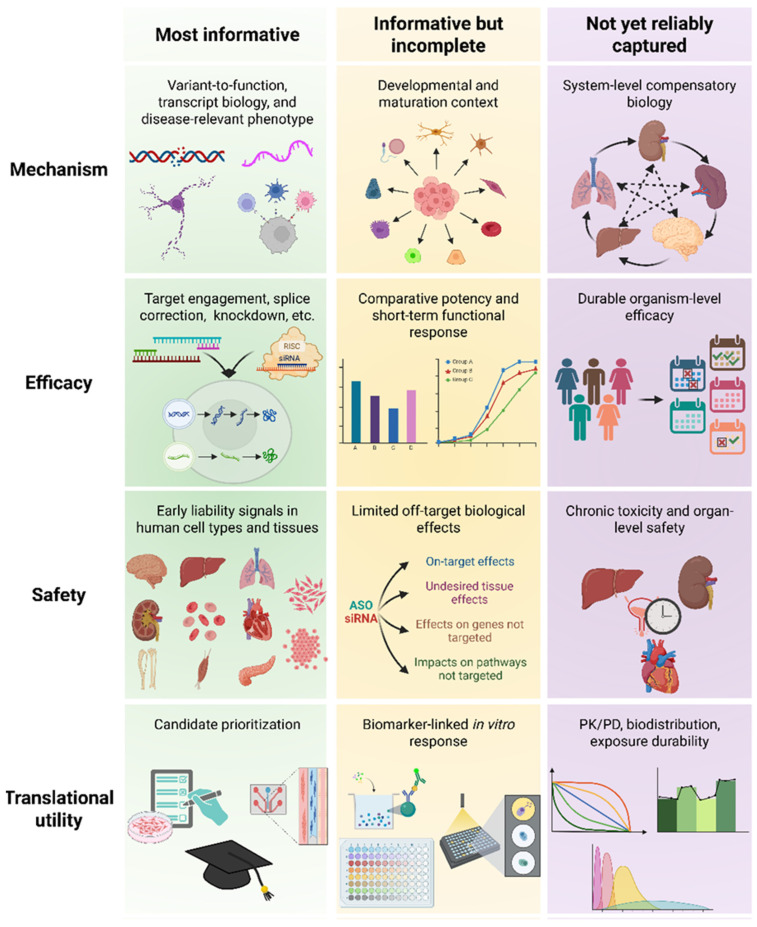
Fit-for-purpose strengths and current boundaries of NAMs in rare-disease RNA therapeutic development. NAMs are most informative when used to address mechanistic and early preclinical questions that depend on disease-relevant human biology, including variant-to-function assessment, transcript-level effects, early efficacy screening, and candidate prioritization. They may also contribute to comparative functional assessment, early safety triage, and biomarker-linked in vitro response, but these functions remain incomplete and context-dependent. By contrast, NAMs do not yet reliably capture organism-level pharmacokinetics, biodistribution, durable whole-body efficacy, chronic toxicology, or broader multicompartment physiology. Here, “informative but incomplete” refers to domains in which NAMs may provide useful partial evidence but cannot support the decision alone. “Not yet reliably captured” refers to domains that generally require organism-level or broader nonclinical evidence, e.g., systemic exposure, biodistribution, chronic toxicology, and durable whole-body efficacy. The figure therefore emphasizes a fit-for-purpose view of NAM utility. Their most defensible current role is as a front-loaded human filter that improves early translational judgment rather than as a universal replacement for animal-based studies. Column colors indicate the proposed level of current NAM utility: green, most informative; yellow, informative but incomplete; purple, not yet reliably captured.

NAMs can also add substantial value during early efficacy screening [45,88,89,90]. For oligonucleotide therapeutics, candidate selection often depends not only on target engagement, but also on splice correction, transcript knockdown, protein restoration, or rescue of a measurable cellular phenotype [45,51]. Human-derived models can provide these readouts in a genotype-matched setting and, in some cases, on timelines compatible with patient-specific development [44,45,75,91]. This creates a realistic opportunity for earlier prioritization of candidates with the strongest mechanistic plausibility [45,75,91]. The clearest current strengths of NAMs lie in questions that depend directly on disease-relevant human biology, particularly variant-to-function assessment, transcript-level effects, early efficacy screening, and candidate prioritization (Figure 2) [31,73,77].

A more cautious, but still important, role for NAMs is in early safety triage [77,78]. These systems may help reveal overt on-target liabilities in vulnerable human cell types, identify major off-target biological effects, and compare candidate oligonucleotides before animal studies begin [45,77,88]. They may therefore improve prioritization and reduce avoidable attrition. However, this role should not be overstated. Current regulatory thinking treats NAMs as fit-for-purpose tools that require technical characterization and validation, not as self-validating substitutes for established nonclinical assessment [32,78,92]. Development still requires careful consideration of clinical pharmacology, immunogenicity, organ impairment, drug interactions, and broader nonclinical safety [16,18,53].

The principal limitations of NAMs arise from what they still capture poorly (Figure 2) [75,77]. Most do not reproduce whole-organism pharmacokinetics, biodistribution, tissue penetration, long-term toxicology, or multicompartment physiology. These gaps are especially important for RNA therapeutics, whose success depends heavily on chemistry, formulation, route of administration, intracellular trafficking, and target-tissue exposure. A NAM may show that an oligonucleotide can work once it reaches the cell of interest [45]. It often cannot show whether that exposure is achievable, durable, and safe in vivo [55,77]. There are also important limits in model fidelity and reproducibility. Human-derived does not automatically mean clinically predictive. Mechanistic rescue in a human-derived system should therefore not be equated with prediction of clinical benefit. A model may correctly show transcript correction or cellular phenotype rescue while still failing to address whether sufficient exposure is achievable in vivo, whether the effect is durable, whether chronic dosing is safe, or whether the measured readout is clinically meaningful. iPSC-derived systems may remain developmentally immature and may not fully capture complex epigenetic, metabolic, or late-onset features of some rare adult-onset conditions [86,93].

As such, standard baseline cultures are often insufficient to model disease biology, requiring researchers to apply exogenous and artificial stressors to induce relevant disease-like state, e.g., epigenetic, metabolic signatures, in reporter lines. Ensuring the forced cellular state faithfully mimics the human disease before initiating any therapeutic screening is critical and often complex. Organoids and engineered tissues may incompletely represent stromal, vascular, neural, or immune interactions [94]. Batch effects, protocol dependence, and variable assay design can further weaken interpretability across laboratories [77,95,96]. For these reasons, the most defensible current role for NAMs is not replacement of animal models, but improvement of early translational judgment. Used carefully, they can strengthen candidate selection, clarify mechanism, and support patient-specific prioritization. However, if used uncritically, they risk creating a false sense of precision. The key question, therefore, is not whether NAMs can do everything, but whether they can do enough, in a fit-for-purpose manner, to improve decisions that matter for rare-disease RNA therapeutic development. That is where their promise remains most credible, and where further validation is most needed.

## 6. Disease Exemplars: Where NAM-Enabled RNA Therapeutics Appear Most Persuasive

The currently available examples remain limited and should not be treated as proof that NAM-enabled pipelines are broadly predictive across rare genetic diseases, rather their value lies elsewhere. Collectively, they assess several distinct translational propositions, including whether molecular diagnosis can be converted into a therapeutic hypothesis within a clinically meaningful timeframe, whether patient-derived models can support preclinical prioritization, and whether human-derived systems can strengthen mechanistic confidence before or alongside animal studies. For this reason, selected exemplars are more informative here than an exhaustive catalogue (Table 1).

### 6.1. Ultra-Rare, Individualized Intervention: Milasen as a Landmark Proof of Principle

The development of milasen remains a landmark example of how molecular diagnosis can be translated into a patient-customized RNA therapeutic on an unusually compressed timeline [44]. In this particular case, identification of a splice-altering intronic variant in *MFSD8*/*CLN7* informed the rational design of a splice-modulating ASO, and proof-of-concept studies in patient-derived cells supported an N-of-1 clinical study within approximately one year of first patient contact [44]. While initial reports indicated objective seizure reduction without serious short-term adverse events, it is imperative to acknowledge the ultimate clinical outcome, i.e., the patient ultimately succumbed to the disease [17,44]. The intervention, while mechanistically precise, was arguably administered “too late” to halt systemic neurodegeneration. The patient-derived cellular studies supported splice correction, but they were not designed to determine whether the disease had already reached a stage at which cellular rescue could translate into meaningful clinical stabilization. This distinction is important because patient-derived models may establish target engagement and molecular rescue without accounting for disease-stage irreversibility, systemic neurodegeneration, or whole-organism trajectory. Even with these limitations, milasen established an important principle: in selected ultra-rare settings, patient-specific mechanistic evidence can support a credible path from diagnosis to therapeutic action when conventional development pathways are unrealistic [44].

### 6.2. Scalable Patient-Derived Screening: Duchenne Muscular Dystrophy Organoids

If milasen provided a proof of principle for patient-specific ASO therapy in an ultra-rare setting, a more recent patient-derived cardiac organoid study in DMD addressed whether elements of this workflow could also be made faster and more scalable [45]. Using cryopreserved peripheral blood mononuclear cells, the investigators established a rapid iPSC reprogramming pipeline and generated patient-specific cardiac organoids for preclinical evaluation of ASO candidates. In a patient carrying a structural *DMD* deletion amenable to exon skipping, ASO treatment restored dystrophin expression and improved disease-associated calcium-handling phenotypes. The platform was then extended to siblings with a deep intronic *DMD* variant, for whom newly designed patient-specific ASOs restored dystrophin expression, corrected splicing, and reversed disease-associated functional abnormalities in cardiac organoids. The reported workflow required approximately 3 weeks to establish iPSC lines and less than 6 weeks of hands-on time from stored patient cells to empirical organoid-based testing. In sum, the findings of this study indicate that patient-derived organoid systems can support rapid, genotype-matched preclinical evaluation in selected rare-disease settings [45]. However, rapid proof-of-concept screening should not be equated with rapid clinical deployment. Translation to patient use remains constrained by delivery, dose selection, toxicology, manufacturing, product quality, regulatory review, and clinically interpretable outcome measures.

### 6.3. Neurodevelopmental Rare Disease: Timothy Syndrome as a Multilevel ASO Rescue Model

Timothy syndrome, a severe genetic disorder caused by a heterozygous gain-of-function variant in the alternatively spliced *CACNA1C* exon 8A, provides another informative example of how RNA therapeutics can be evaluated across multiple human-derived disease models within a single mechanistic framework [97,98,99]. In a recent study, ASOs were developed to suppress exon 8A inclusion and shift exon usage toward exon 8 [89]. The ASO-mediated splice switch rescued disease-relevant abnormalities in patient-derived cortical organoids and restored interneuron migration defects in forebrain assembloids. The investigators then extended the analysis to an in vivo transplantation setting and reported that a single intrathecal ASO administration reduced exon 8A expression and rescued calcium-related and dendritic phenotypes in transplanted human neurons. The findings of this study indicate that human-derived organoid and assembloid systems can support mechanistically precise ASO evaluation in a neurodevelopmental rare disease and can be integrated with downstream in vivo validation when the question extends beyond cell-autonomous rescue [89].

### 6.4. Rare Diseases with Cardiovascular Involvement: Calmodulinopathy as a Mechanistic Test Case

Calmodulinopathy provides a unique mechanistic context in which the utility of NAMs can be examined. These rare inherited arrhythmia syndromes arise from heterozygous variants in *CALM1*, *CALM2*, or *CALM3*, three genes that encode identical calmodulin protein [100,101,102]. This genetic architecture creates a therapeutic setting in which selective depletion of the affected gene may reduce the pathogenic transcript while preserving overall calmodulin through the remaining paralogues. A recent study took advantage of variant-bearing human iPSC-derived cardiomyocytes, to demonstrate that *CALM1* knockout or *CALM1*-depleting ASOs did not reduce total calmodulin protein levels and normalized repolarization [90]. In a complementary mouse model, an ASO targeting murine *Calm1* reduced transcript levels and attenuated drug-induced ventricular tachycardia without detectable deleterious effects on cardiac electrical or contractile function. Taken together, the human iPSC-cardiomyocyte and mouse data support a layered, sequential translational workflow in which mechanistic evidence was generated in a human-derived system and in vivo functional context was evaluated in the animal model [90]. In this setting, the study is more consistent with a complementary than a substitutive role for NAMs.

### 6.5. What These Exemplars Suggest, and What They Still Do Not Prove

These example studies suggest that NAM-enabled RNA therapeutic development is most persuasive when several conditions align, including (but not limited to): the disease mechanism is genetically well defined, the therapeutic hypothesis is transcript-centered, the phenotype can be measured in a relevant human cellular context, and the preclinical question is one of mechanistic plausibility or candidate prioritization rather than organism-level prediction [44,45,89,90]. The examples also support different levels of inference. Milasen primarily illustrates individualized proof of principle in an ultra-rare setting. The DMD and Timothy syndrome studies support mechanistically rich patient-derived screening pipelines and phenotype rescue in human-derived systems. The calmodulinopathy study illustrates a more layered translational architecture, in which human iPSC-derived cardiomyocytes provided mechanistic evidence and the animal model provided in vivo functional context. These examples should therefore not be treated as equivalent forms of evidence. Simultaneously, the boundaries of inference remain clear. These examples do not show that NAM-guided pipelines are broadly generalizable across rare genetic diseases. They do not eliminate the need for in vivo pharmacology, biodistribution, toxicology, or longer-term clinical evidence. Nor do they establish that apparent rescue in a patient-derived model will reliably predict patient benefit across tissues and time scales. What they do show is narrower, but still important: in selected settings, human-derived systems can generate decision-relevant evidence that conventional pipelines often fails to provide quickly or specifically enough. That narrower claim is also the more defensible one.

## 7. Translational Realities Beyond Proof-of-Concept

Successful demonstration of proof of concept for therapeutic strategies is often not the main bottleneck in rare-disease RNA therapeutics. Pediatric age adds another layer of translational complexity. Many rare genetic diseases present early in life, and developmental stage, tissue maturation, growth, age-dependent pharmacology, and narrow therapeutic windows may influence both model choice and interpretation of efficacy or safety signals. In pediatric or rapidly progressive disorders, a NAM is most useful when it reflects the disease-relevant developmental context and generates interpretable data within a clinically meaningful timeframe. The more puzzling question is whether a mechanistically persuasive candidate can be translated into a therapy that is deliverable, interpretable, scalable, and clinically usable. This is where many promising programmes slow down. In rare genetic diseases, the path from sequence design to patient benefit is constrained not only by biology, but also by chemistry, tissue access, pharmacology, manufacturing, regulation, and affordability [16,18,19]. RNA therapeutics therefore require a modular translational framework that extends beyond target engagement alone.

The foremost constraints are the optimization of chemistry and the delivery strategy. Oligonucleotide performance depends heavily on backbone chemistry, sugar modification, conjugation strategy, formulation, and route of administration [12,19,72,103,104,105]. These variables influence stability, potency, protein binding, intracellular trafficking, and toxicity, and they often determine whether a mechanistically attractive ASO is clinically viable. Some tissues remain comparatively accessible, particularly the liver and, through intrathecal administration, parts of the central nervous system [12,103,104]. By contrast, efficient extrahepatic delivery remains a major translational limitation, especially for tissues, e.g., heart and skeletal muscle. Recent efforts to improve delivery to muscle reinforce the same point that delivery remains one of the principal determinants of success. Additional barriers exist between sequence design and effective tissue-level action, including but not limited to tissue penetration, receptor- or ligand-dependent uptake, endosomal escape, intracellular trafficking, protein-binding and, where relevant, protein-corona effects, and chemistry-dependent immune or toxicity liabilities. GalNAc conjugation has substantially improved hepatocyte-directed delivery, but this success does not generalize automatically to extrahepatic tissues [55,106]. Delivery to skeletal muscle, heart, and some central nervous system indications remains more difficult and may require additional chemistry, conjugation, nanoparticle shuttle formulation, or local administration strategies [69,107,108,109,110]. These considerations reinforce that RNA tractability at the sequence level does not by itself establish translational feasibility.

Current patient-derived cell-based in vitro models and animal models often fail to efficiently predict the impact of chemistry and delivery optimization efforts efficiently. One response to these translational gaps could be and has been the development of hybrid organoid-on-chip and multiorgan microphysiological systems [111,112]. By combining self-organizing human tissues with controlled perfusion, mechanical cues, and, in some cases, interconnected organ circuits, these platforms begin to narrow the space between cell-autonomous rescue and organism-level inference. Organoid-on-chip systems can improve physiological control relative to static culture, and multiorgan circuits can incorporate vascular flow and inter-tissue communication [111,112]. These advances do not remove the limits of NAMs, but they suggest that the relevant question is increasingly one of required physiological depth rather than simple platform category.

A second constraint is pharmacology and safety interpretation. Oligonucleotide therapeutics present nonclinical challenges that differ in important ways from those of small molecules or therapeutic proteins [16,18,19]. Sequence-dependent and chemistry-dependent liabilities, immunostimulatory effects, organ-specific accumulation, class-related toxicities, and uncertainties around chronic dosing all complicate development [19]. NAMs may strengthen early mechanistic judgment and support candidate prioritization; however, they do not yet capture whole-organism pharmacokinetics, biodistribution, multicompartment exposure, or long-term toxicology with sufficient robustness [31,32,92]. As such, the most credible role for NAMs in translation remains selective and fit-for-purpose: they can sharpen early decisions, but they do not remove the need for broader nonclinical evidence. Safety considerations also vary by oligonucleotide class, chemistry, dose, route, and tissue exposure. Depending on the product, reported or anticipated concerns may include hybridization-dependent off-target effects, exaggerated on-target effects, innate immune activation, complement or inflammatory responses, thrombocytopenia or coagulation-related findings for some chemistries, renal and hepatic accumulation, injection- or infusion-related reactions, and organ-impairment or drug-interaction considerations [113]. These risks are not uniform across all RNA therapeutics and should therefore be assessed in a product-specific manner rather than inferred from sequence specificity alone [105,114].

A third constraint is manufacturing and timeline feasibility. One attraction of RNA therapeutics is design speed. However, rapid design does not automatically translate into rapid clinical readiness. Analytical characterization, synthesis, purification, formulation, batch release, and quality control can all become limiting steps, especially when development is individualized or low-volume [12,103,104,115]. Studies on alternative oligonucleotide synthesis approaches point at the constraints of current manufacturing process, including scalability, solvent burden, throughput, and sustainability. In rare genetic disease, where therapeutic windows may be narrow and patient numbers are small, these practical issues are not secondary; rather they directly influence whether individualized development is realistic.

A fourth constraint is regulatory fit. Regulatory thinking has evolved substantially. The U.S. Food and Drug Administration (FDA) now has draft guidance both for individualized ASO development in severely debilitating or life-threatening genetic diseases and for the broader use of NAMs in drug development [16,18,32,92]. At the same time, these documents do not support a simplified pathway based on human-derived models alone [32,92]. Instead, they emphasize early interaction with the Agency, fit-for-purpose nonclinical programmes, and evidence that is adequate for the specific regulatory question being asked. In that sense, the regulatory landscape has become more permissive toward individualized and NAM-supported development, but it remains evidence-driven rather than technology-driven.

Last but not least, access and equity should be treated as translational issues rather than postscript concerns. Rare diseases affect more than 300 million people globally, and the World Health Assembly of the World Health Organization (WHO) has now formally recognized rare diseases as a global health priority [116]. At the same time, inequitable access to rare-disease diagnostics and therapies remains a major challenge across and within countries [116,117]. This is directly relevant here because highly individualized RNA therapies risk deepening existing inequities if their development pathways remain technically feasible but economically inaccessible. A translational model cannot be considered mature if it improves therapeutic nomination while leaving manufacturing, affordability, and real-world access unresolved.

These realities do not weaken the case for RNA therapeutics or for NAMs. They define the conditions under which that case becomes credible. The most realistic path forward is neither to treat proof-of-concept rescue in a human-derived model as sufficient, nor to dismiss such evidence as marginal. Rather, it is to embed mechanistic, human-relevant data within a broader translational framework that includes delivery strategy, pharmacologic interpretability, manufacturability, regulatory usability, and equitable access. That broader framework will determine whether the path from molecular diagnosis to individualized therapy can become operational at scale.

## 8. Regulatory Pathways and Evidentiary Expectations for Individualized RNA Therapies

The regulatory challenge in individualized RNA therapeutics is not simply rarity. It arises from the mismatch between conventional evidentiary models and therapies designed for one patient, one family, or an exceedingly small molecularly defined subgroup. The FDA’s draft guidance on individualized ASOs was written specifically for severely debilitating or life-threatening genetic diseases in which the treatment-amenable variant is unique to, or found in, only one or two patients [16,18]. The same guidance also makes it clear that, once more than a few patients are candidates for a product, the programme should no longer be treated as individualized and should instead follow a more conventional development pathway. It is important to distinguish regulatory direction from operationalized regulatory pathways. At the time this review was prepared, several FDA documents relevant to this discussion remained draft or preliminary or otherwise non-final guidance only, including those addressing individualized ASO clinical recommendations, individualized ASO nonclinical testing, individualized ASO CMC, the plausible mechanism framework for individualized therapies, and general considerations for NAM use in drug development [16,18,19,32,92]. By contrast, FDA’s clinical pharmacology guidance for oligonucleotide therapeutics is final guidance [18,19]. Therefore, these documents should be interpreted as current regulatory thinking and evidentiary direction rather than as standardized pathways that guarantee development or approval of individualized RNA therapeutics.

Recent FDA guidance indicates that the evidentiary framework for such products is becoming more explicit, but not looser [118]. For RNA therapeutics, the February 2026 draft guidance on the plausible mechanism framework implies a practical and highly structured evidentiary hierarchy. First and foremost, the therapeutic strategy must be able to demonstrate strict biological relevance and a tight linkage to the specific disease-causing variant. Second, the nonclinical programme must be fit-for-purpose, providing sufficient proof-of-concept and adequate safety data to justify clinical use. For individualized ASOs specifically, US FDA nonclinical draft guidance recommends development within a well-characterized chemical class supported by substantial prior nonclinical and clinical experience [16]. Recognizing that randomized controlled trials are often not feasible in rare disease settings, development must be malleable and exploit alternative trial designs, e.g., externally controlled investigations, baseline lead-in periods, and robust natural history data.

The expected clinical evidence is also more structured than is sometimes assumed. The US FDA recommends standardized and prespecified assessments of clinical efficacy, safety outcomes, and relevant biomarkers [118]. A substantial clinical improvement that is distinctly inconsistent with the untreated natural history may only contribute to evidence of effectiveness if alternative explanations can be reasonably excluded. Within this framework, biomarkers serve a critical utility at several levels, supporting proof-of-concept, target engagement, safety monitoring, and dose selection. Importantly, the guidance distinguishes between biomarkers that function as validated surrogates for traditional approval and those serving as reasonably likely predictors for accelerated approval, a critical distinction that is indispensable in rare disease settings where direct clinical change is often slow to emerge and/or difficult to quantify.

NAMs fit into this framework as supportive and context-dependent evidence rather than as stand-alone regulatory substitutes. The FDA’s March 2026 draft guidance on NAMs provides a validation framework centred on context of use, human biological relevance, technical characterization, and fit-for-purpose performance [32,92]. It also reflects the post-2022 regulatory environment in which nonanimal approaches may, in appropriate circumstances, contribute to Investigational New Drug (IND)-supporting submissions. At the same time, the guidance does not endorse generic substitution of NAMs for animal studies. Its emphasis is on validation principles, study design, and evidentiary fitness for a defined use case. In regulatory terms, the most credible use of NAMs is therefore selective: to strengthen mechanistic plausibility, refine candidate selection, and support specific decision points within a broader evidentiary package. Accordingly, NAMs should be interpreted as potential contributors to a weight-of-evidence package rather than as broadly validated substitutes for animal studies.

Chemistry, Manufacturing, and Controls (CMC) expectations further reinforce that individualized development is not exempt from rigour. The FDA’s plausible mechanism framework states that individualized therapies must still be manufactured to regulatory quality standards and that CMC development should proceed alongside clinical development [18,118]. The individualized ASO CMC guidance likewise emphasizes defined product attributes, appropriate controls, reliance on prior knowledge where justified, and minimization of manufacturing changes that could introduce comparability problems. For individualized RNA therapeutics, this means that the regulatory bottleneck is not only clinical evidence, but also whether product quality, consistency, and shelf-life strategy are sufficiently mature for meaningful clinical use.

Taken together, current regulatory thinking does not support a simplified narrative in which individualized RNA therapeutics proceed on mechanistic enthusiasm alone. At the same time, it does not impose evidentiary expectations that make ultra-rare development impossible, by definition. Rather, it points toward a narrower and more practical model: strong mechanistic rationale, fit-for-purpose nonclinical evidence, disciplined outcome assessment, careful use of natural history and biomarkers, early regulatory engagement, and product quality proportionate to the intended clinical use. That is the evidentiary space in which NAM-enabled individualized RNA therapeutics may become more practically assessable, if evidence remains fit-for-purpose, product-specific, and shaped through early interaction with the Agency.

## 9. A Pragmatic Validation Framework for NAMs in Rare Genetic Diseases

**Figure 3:**
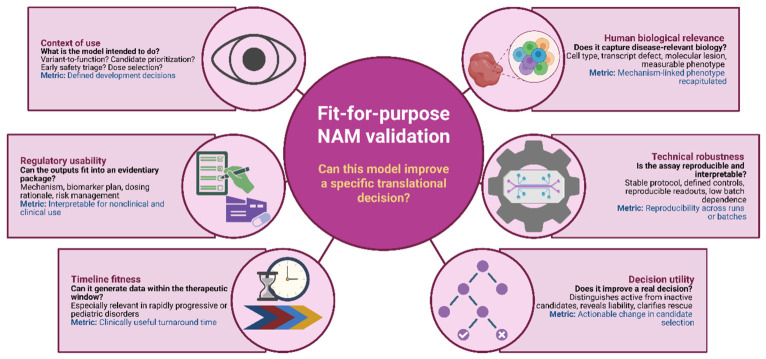
A pragmatic validation framework for NAMs in rare-disease RNA therapeutic development. Validation of a NAM should not be treated as a binary designation. In rare genetic diseases, the key question is whether a given NAM is sufficiently reliable, relevant, and interpretable for a specific translational purpose. This framework organizes validation into six linked domains: context of use, human biological relevance, technical robustness, decision utility, timeline fitness, and regulatory usability. Together, these domains provide a fit-for-purpose basis for evaluating whether a NAM can credibly inform mechanistic interpretation, candidate prioritization, or other decisions in RNA therapeutic development. The framework does not require NAMs to replace organism-level pharmacology, biodistribution, or chronic toxicology. Instead, it defines the narrower conditions under which NAM-derived evidence becomes scientifically defensible and decision-relevant.

Validation should not be viewed as an all-or-none label. A NAM does not become broadly credible simply because it is human-derived, nor does it become unusable because it cannot replace every function of an animal model [32,119,120]. In rare genetic diseases, the more relevant question is whether a given NAM is sufficiently reliable, relevant, and interpretable for a specific development decision [32]. This view is consistent with the current thinking of the FDA, which emphasizes validation within a defined context of use and recognizes that even non-validated NAMs may still contribute within a fit-for-purpose, weight-of-evidence framework [32,120]. A practical validation framework for NAMs in rare-disease RNA therapeutic development can be organized into six linked domains: context of use, human biological relevance, technical robustness, decision utility, timeline fitness, and regulatory usability (Figure 3, Table 2). To note, these domains should not be taken into consideration as independent checkboxes. Rather, they are interrelated and define the conditions under which NAM-derived evidence becomes scientifically interpretable and translationally useful when considered together.

First, the context of use should be defined explicitly [32]. A credible validation framework must begin by stating exactly what the NAM is intended to do. It may be used to establish variant-to-function causality, prioritize among ASO candidates, support dose selection, investigate a mechanistic toxicity signal, or justify why a given animal model may not be sufficiently informative. A model that is informative for one of these purposes may be inadequate for another. Current guidance from the FDA is explicit on this point: usefulness depends on whether the method addresses a defined data gap and supports a specific regulatory or drug-development decision [32]. This distinction is important because different model purposes carry different evidentiary expectations. A NAM used for mechanistic clarification or candidate ranking does not serve the same evidentiary function as a model intended to predict clinical efficacy, chronic toxicity, or long-term safety. The intended context of use should therefore determine the level and type of validation required.

Second, human biological relevance should be demonstrated empirically rather than assumed [32,120]. For rare-disease RNA therapeutics, this means showing that the model reproduces the disease-relevant cell type, transcript biology, molecular lesion, and measurable phenotype most likely to matter for therapeutic interpretation. In practice, that may require evidence that the model reproduces abnormal splicing, toxic transcript accumulation, protein deficiency, electrophysiologic dysfunction, or another mechanism-linked phenotype that is plausibly connected to clinical disease. Human derivation alone is not sufficient. The model should reproduce the specific biology that the oligonucleotide-based strategy is intended to modify.

Third, technical robustness and reproducibility should be treated as core validation criteria rather than supplementary details [32]. A NAM cannot be considered decision-ready if assay conditions, cell sources, differentiation protocols, controls, readouts, or analytical pipelines are poorly defined or unstable across runs [95,96]. This is especially important for patient-derived systems, in which donor variability, developmental immaturity, and batch effects may materially influence results. The FDA’s NAM guidance places strong emphasis on technical characterization, including clear documentation of assay methods, controls, cell source, variability, and performance within the intended context of use [32,120].

Fourth, decision utility should be assessed directly [32]. In many rare diseases, there will be no large benchmark dataset against which classical performance metrics can be calculated at scale. Even then, validation should still ask whether the model improves a real translational decision, and if it is able to distinguish active from inactive candidates, identify an on-target liability that changes candidate selection, and clarify whether a phenotype rescue is mechanistically meaningful rather than incidental [32]. In rare-disease RNA therapeutics, this decision-centred view is often more realistic than a claim of universal predictive validity.

Fifth, timeline fitness should be considered part of validation in individualized settings [121]. A technically sophisticated model that requires several months to establish may be less useful in a rapidly progressive pediatric disease than a simpler system that yields interpretable data within weeks. This does not mean that speed should override rigour. Rather, it means that validation in rare-disease precision medicine should include whether a NAM can generate actionable evidence within the therapeutic window relevant to the disease. This consideration is implicit in current regulatory thinking around individualized therapies, where evidence must not only be sufficient, but also obtainable within a clinically meaningful timeframe [16,18,118,122].

Sixth, regulatory usability should be built into validation from the outset [92]. A NAM may be biologically compelling yet still fail to influence development if its outputs are not interpretable within a regulatory evidence package. The most useful NAMs will therefore be those whose endpoints can be linked to product mechanism, biomarker strategy, dose rationale, safety interpretation, or nonclinical justification. In this sense, validation is not only about scientific performance. It is also about whether the data generated by the model can be incorporated into the chemistry, nonclinical, and clinical logic of an individualized therapeutic programme.

This framework also implies that validation cannot remain specific to individual laboratory settings. Broader translational use will require shared benchmarking standards, transparent technical reporting, and cross-platform qualification efforts. Recent efforts in this direction emphasize reproducibility, protocol standardization, and fit-for-purpose development of human-based systems [120,123,124]. These developments support a view of validation that is operational and comparative, rather than merely conceptual.

As a practical example of how this six-domain framework could be applied, a patient-derived iPSC-cardiomyocyte model used to prioritize ASO candidates for a rare arrhythmia disorder would first require a defined context of use, such as candidate ranking rather than prediction of long-term clinical efficacy. Human biological relevance would require reproduction of the mutation-linked electrophysiological phenotype and the transcript target of the ASO. Technical robustness would require reproducible differentiation, prespecified electrophysiological readouts, and appropriate positive and negative controls. Decision utility would be shown if the model separates active from inactive ASOs or changes candidate ranking. Timeline fitness would depend on whether the assay can generate interpretable data within the clinical decision window. Regulatory usability would require that the endpoints can be linked to mechanism, dose rationale, biomarker strategy, or nonclinical justification.

Overall, these present a pragmatic validation framework with six linked domains, i.e., defined context of use, demonstrated human biological relevance, technical robustness, decision utility, timeline fitness, and regulatory usability. Such a framework is intentionally narrower than a claim of universal predictivity. It does not ask a NAM to replace organism-level pharmacology, biodistribution, or chronic toxicology when it cannot. Instead, it asks whether the model is credible enough to improve a specific translational decision in a setting where conventional evidence is often incomplete. That narrower standard is also the more defensible one. It allows progress without lowering the threshold for scientific rigour.

## 10. Conclusions and Future Directions

Rare genetic diseases provide foundational settings in which to examine the promise and the limits of precision medicine. Molecular diagnosis can now often be established with far greater precision than therapeutic action. RNA therapeutics offer a plausible way to narrow that gap because they can be designed against defined transcript-level mechanisms. However, their success depends not only on sequence design, but also on the biological systems used to evaluate them. In this context, NAMs have emerged as potentially useful, but still incomplete, tools. Their value does not lie in replacing animal models in a universal sense, but rather, in their potential to function as a front-loaded human filter that can better clarify mechanism, improve candidate prioritization, and strengthen early translational judgment in settings where conventional development pathways are often slow, imprecise, or impractical. However, important limitations remain, particularly in relation to pharmacokinetics, biodistribution, long-term safety, tissue complexity, and reproducibility. These limitations should not be minimized. The central question, therefore, is not whether NAMs are inherently transformative or inherently insufficient, but whether they can be validated rigorously enough to improve specific development decisions for the development of RNA therapeutics for rare diseases. A pragmatic framework based on context of use, biological relevance, technical robustness, decision utility, timeline fitness, and regulatory usability provides a more realistic basis for addressing that question than broad claims of disruption or replacement.

A plausible next phase will involve tighter integration of patient-derived cellular systems, microphysiological platforms, and computational methods into iterative development workflows. In selected settings, such integration may support faster hypothesis testing, more informed candidate refinement, and better alignment between mechanistic evidence and downstream translational decision-making. Whether such workflows become broadly useful, however, will depend on the same conditions emphasized throughout this review: fit-for-purpose validation, disciplined interpretation, and realistic understanding of what each model can and cannot contribute. The path from molecular diagnosis to individualized therapy will not become credible through technological enthusiasm alone, but will depend on rigorous model validation, careful integration of human-derived evidence and in vivo evidence, regulatory alignment, and sustained attention to manufacturability and access. Under those conditions, NAM-enabled RNA therapeutic development may become not a conceptual exception, but a practical component of rare-disease translational medicine. The practical goal is not to replace every preclinical model, but to use the right model for the right decision at the right stage of development.

## Figures and Tables

**Table 1:** Representative examples of NAM-enabled RNA therapeutic development in rare genetic diseases. This table is selective and illustrative rather than exhaustive and the examples included were selected to represent distinct translational roles for NAMs in rare-disease RNA therapeutic development.

Disease/Condition	Gene/Molecular Lesion	RNA Therapeutic Strategy	Platform	What the NAM Contributed	Key Translational Insight	Major Limitations That Remained
*CLN7* Batten disease (milasen)	*MFSD8*/*CLN7* splice-altering intronic variant	Patient-customized splice-modulating ASO	Patient-derived cells	Proof-of-concept for variant-specific splice correction before N-of-1 clinical use	Molecular diagnosis can, in selected ultra-rare settings, be converted into individualized RNA therapy on a compressed timeline	Does not by itself establish broad predictive validity or resolve long-term efficacy and safety
Duchenne muscular dystrophy	Exon-skipping-amenable structural variant in *DMD*	Exon-skipping ASO	Patient-derived iPSCs and cardiac organoids	Genotype-matched testing of dystrophin rescue and functional phenotype improvement	Human-derived organoid systems can support rapid preclinical candidate screening and mechanistic prioritization	Does not resolve systemic delivery, chronic toxicity, or full regulatory sufficiency
Duchenne muscular dystrophy	Deep intronic *DMD* splice defect	Patient-specific splice-correcting ASOs	Patient-derived iPSCs and cardiac organoids	Design and empirical testing of novel patient-specific ASOs	NAMs may help shorten the path from deep-variant diagnosis to therapeutic nomination in selected settings	Organism-level PK, biodistribution, and durability remain outside the model’s reach
Timothy syndrome type 1	*CACNA1C* exon 8A gain-of-function variant (p.G406R)	Splice-switching ASO to reduce exon 8A inclusion and favor exon 8 usage	Patient-derived cortical organoids, forebrain assemblies, and transplantation-based in vivo extension	Mechanistic evaluation of ASO-mediated splice correction and rescue of disease-relevant neural phenotypes	Human-derived neural NAMs can support multilevel mechanistic testing of ASOs in rare neurodevelopmental disease and can be linked to downstream in vivo validation	Does not by itself establish clinical efficacy, long-term safety, or broader regulatory sufficiency
Calmodulinopathy	Heterozygous variants in *CALM1*/*CALM2*/*CALM3*	Gene-selective ASO depletion	Human iPSC-derived cardiomyocytes, paired with mouse in vivo studies	Disease-relevant mechanistic evidence that selective depletion could normalize repolarization without reducing total calmodulin protein	NAMs are particularly useful when integrated into a layered translational package rather than treated as stand-alone replacements	In vivo context remained necessary for arrhythmia-level functional and safety interpretation

**Table 2:** Pragmatic validation domains for NAM use in rare-disease RNA therapeutic development. The framework is intentionally narrower than a claim of universal predictivity. Its purpose is to define when a NAM is sufficiently reliable, relevant, and interpretable to support a specific translational decision.

Validation Domain	Core Question	What Should Be Shown	Example Indicator(s)	Common Reason for Failure
Context of use	What specific decision is the NAM intended to inform?	Explicit statement of whether the model is being used for variant-to-function analysis, candidate prioritization, early safety triage, dose exploration, or another defined purpose	Prespecified development question; defined decision point	Model is presented as generally useful without a clearly bounded purpose
Human biological relevance	Does the model capture disease-relevant biology?	Evidence that the model reflects the relevant cell type, transcript abnormality, molecular lesion, and mechanism-linked phenotype	Abnormal splicing, toxic transcript accumulation, protein deficiency, electrophysiologic dysfunction, or another disease-relevant phenotype reproduced	Human-derived system lacks the key biology the therapeutic is intended to modify
Technical robustness	Is the assay reproducible and interpretable?	Stable protocol, defined controls, reproducible readouts, and manageable batch variability	Concordant results across runs, batches, operators, or cell preparations	Signal depends heavily on protocol drift, donor effects, or unstable differentiation quality
Decision utility	Does the model improve a real translational decision?	Evidence that the NAM meaningfully changes candidate ranking, liability recognition, or mechanistic interpretation	Separation of active vs. inactive candidates; identification of a liability that alters prioritization	Model generates data but does not alter any practical development decision
Timeline fitness	Can the model generate useful data within the therapeutic window?	Turnaround time compatible with the disease context, especially in ultra-rare, pediatric, or rapidly progressive settings	Time from sample acquisition to actionable readout	Model is biologically elegant but too slow to influence therapeutic action
Regulatory usability	Can the outputs fit into a broader evidentiary package?	Endpoints that can be linked to mechanism, biomarker strategy, dosing rationale, safety interpretation, or nonclinical justification	Readouts interpretable within CMC, nonclinical, biomarker, or clinical planning logic	Data are biologically interesting but difficult to use in regulatory or translational decision-making

## Data Availability

No new data were generated or analyzed in this study. The original contributions presented in this review are included in the article. Further inquiries can be directed to the corresponding author.

## Abbreviations

The following abbreviations are used in this manuscript:

ASO	Antisense Oligonucleotide
CMC	Chemistry, Manufacturing, and Controls
CTiD	Clinical-Trials-in-a-Dish
DMD	Duchenne Muscular Dystrophy
EU	European Union
hATTR	Hereditary Transthyretin-Mediated Amyloidosis
IND	Investigational New Drug
iPSC	Induced Pluripotent Stem Cell
NAM	New Approach Methodology
PK/PD	Pharmacokinetics/Pharmacodynamics
siRNA	Small Interfering RNA
SMA	Spinal Muscular Atrophy
U.S. FDA	United States Food and Drug Administration
WHO	World Health Organization
